# Preventing chemotherapy-induced alopecia: a prospective clinical trial on the efficacy and safety of a scalp-cooling system in early breast cancer patients treated with anthracyclines

**DOI:** 10.1038/s41416-019-0520-8

**Published:** 2019-07-15

**Authors:** Elisabetta Munzone, Vincenzo Bagnardi, Giuseppe Campennì, Ketti Mazzocco, Eleonora Pagan, Andrea Tramacere, Marianna Masiero, Monica Iorfida, Manuelita Mazza, Emilia Montagna, Giuseppe Cancello, Nadia Bianco, Antonella Palazzo, Anna Cardillo, Silvia Dellapasqua, Claudia Sangalli, Greta Pettini, Gabriella Pravettoni, Marco Colleoni, Paolo Veronesi

**Affiliations:** 1 Division of Medical Senology, European Institute of Oncology, IRCCS, Milan, Italy; 2 Department of Statistics and Quantitative Methods, University of Milan-Bicocca, Milan, Italy; 3 Department of Oncology and Hemato-Oncology, University of Milan and Applied Research Division for Cognitive and Psychological Science, European Institute of Oncology, IRCCS, Milan, Italy; 4 Day Hospital, European Institute of Oncology, IRCCS, Milan, Italy; 5 Department of Biomedical and Clinical Sciences, University of Milan and Applied Research Division for Cognitive and Psychological Science, European Institute of Oncology, Milan, Italy; 6 Data Management, European Institute of Oncology, IRCCS, Milan, Italy; 7 Program of Breast Health, European Institute of Oncology, IRCCS, Milan, Italy; 8 Department of Oncology and Hemato-Oncology, University of Milan, Milan, Italy

**Keywords:** Breast cancer, Skin manifestations

## Abstract

**Background:**

Chemotherapy-induced alopecia (CIA) is a distressing side effect of cancer therapy. The trial aimed to assess feasibility and effectiveness of scalp-cooling system DigniCap® to prevent CIA in primary breast cancer patients receiving an anthracycline containing adjuvant chemotherapy (CT).

**Methods:**

Hair loss (HL) was evaluated by patient self-assessment and by the physician according to the Dean’s scale at baseline and after each cycle of CT. The primary efficacy endpoint was the patient self-assessment HL score evaluated at least 3 weeks after completing CT. A Dean's scale score of 0–2 (i.e. HL ≤50%) was considered a success.

**Results:**

From July 2014 to November 2016, 139 consecutive breast cancer patients were enrolled and received at least one treatment with scalp cooling. Fifty-six out of 131 evaluated patients successfully prevented HL (43%, 95% CI: 34–51%). Twenty-four patients (32%) discontinued the scalp cooling because of alopecia or scalp-cooling related AE, three patients had missing information on CIA, and 48 patients (64%) had a HL greater than 50% after CT. No serious AEs were reported.

**Conclusions:**

DigniCap® System resulted as a promising medical device to be safely integrated in supportive care of early breast cancer patients. Longer follow-up is needed to assess long-term safety and feasibility.

**Clinical trial registration number:**

NCT03712696.

## Background

Breast cancer diagnosis is psychologically distressing, and decisions about adjuvant therapy usually increase this distress. Alopecia is a non-life threatening but disturbing side effect of almost all effective adjuvant chemotherapy regimens for breast cancer. There are many toxicities associated with chemotherapy, but alopecia is the most public stigma of this treatment and it may represent a real deterrent for many patients.^1,2^

The degree of alopecia varies among different drugs. Chemotherapeutic agents, such as the taxanes and anthracyclines, are associated with significant alopecia as one of their main side effects. The percentage of patients experiencing alopecia associated with the use of anthracyclines or taxanes is over 70% in most studies.^3^ Alopecia induced by these agents is dose dependent, although its severity also depends on the combination of other cytotoxic agents.^4^ Chemotherapy-induced alopecia (CIA) affects quality of life and potentially impacts decisions regarding the risks and benefits of treatment.^5^

Scalp-cooling devices are certainly the most well-studied and published technique for preventing CIA, dating back to the 1970s. Scalp hypothermia is hypothesised to prevent CIA by reducing blood perfusion and therefore chemotherapy delivery to the scalp, reducing intrafollicular metabolism.^6^ Several randomised and non-randomised clinical trials have reported cooling results in hair preservation, with discordant conclusions due to differences in chemotherapy regimens, patient populations, hair-loss evaluation methods and cooling mechanisms.^7^

Newer self-contained technologies use a machine to cool and circulate a glycol-based fluid in channels within a cap, allowing the scalp temperature to be controlled and maintained throughout the treatment course. These systems include the DigniCap® Scalp Cooling System (Dignitana, Sweden), that is a medical device developed to provide continuous scalp cooling during chemotherapy infusion. In this prospective two-stage design clinical trial, we sought to assess the feasibility and the effectiveness of scalp-cooling system DigniCap® to prevent alopecia in early breast cancer patients receiving adjuvant chemotherapy with either anthracycline and/or taxanes.

## Methods

### Study design

Early breast cancer patients undergoing adjuvant chemotherapy at European Institute Oncology of Milan- Italy were assessed for participating into this prospective trial evaluating the role of the DigniCap® system for the prevention of CIA. The study was approved by the local ethic committee and was performed in accordance with the Declaration of Helsinki. All patients signed written informed consent.

### Patients

Patients with a documented diagnosis of early-stage breast cancer and with a planned course of chemotherapy in the adjuvant setting with curative intent were eligible. Eligible chemotherapy regimens included at least one of the following:

- Doxorubicin 60 mg/m2 or Epirubicin 90 mg/m2 and cyclophosphamide 600 mg/m2 for four cycles IV every 3 weeks followed or not by Paclitaxel 80 mg/m2 weekly IV for at least 12 weeks or Docetaxel 75–100 mg/m2 IV every 3 weeks for four cycles with or without IV trastuzumab
- Docetaxel 75 mg/m2 and cyclophosphamide 600 mg/m2 for four cycles IV every 3 weeks
- Paclitaxel 80 mg/m2 weekly IV for at least 12 weeks *or* Docetaxel 75–100 mg/m2 IV every 3 weeks for four cycles with or without IV trastuzumab

Major exclusion criteria were Performance status (ECOG) 2 or higher, previous chemotherapy, autoimmune disease affecting hair (e.g. alopecia areata), history of whole brain radiation or cold agglutinin disease or cryoglobulinemia.

A clinical assessment of all patients was performed at baseline, at the end of each cycle of chemotherapy, and at the end of the planned chemotherapy treatment. The DigniCap® System was used at each cycle on the day of chemotherapy administration and a primary nurse was dedicated to patients enrolled in the trial.

### Scalp-cooling procedures

The scalp-cooling period initiated ~30 min before chemotherapy, the administration of cytostatics started when the temperature reached the desired level between 3 °C and 5 °C. An individually sized silicone cap was kept on the head of the patient continuously throughout chemotherapy infusion period. Any deviations were registered and corrected by the feedback provided to the system by the sensors. Scalp temperature was monitored by two separate sensors at the front and back of the cap. An additional sensor ensures that the temperature never decreases below freezing. Deviations from the default temperature were automatically adjusted. Scalp temperature was to be maintained at 3–5 °C throughout chemotherapy and for 90 min to 120 min afterward, depending on the chemotherapy drug and dose, resulting in a scalp temperature of ~15 °C.^8^

### Assessment of hair loss

Hair loss (HL) was evaluated by patient self-assessment and by the medical personnel according to the Dean’s scale [grade 0 (no HL), 1 (<25% HL), 2 (25–50% HL), 3 (50–75% HL) and 4 (>75% HL)] by means of five standardised photographs taken prior to each chemotherapy cycle and at the end of planned treatment.

Photographs of patient’s head were taken from five different views: frontal, right and left lateral, occipital and crown. To assess hair status, photographs of patients were taken by study personnel before the start of each chemotherapy cycle and at 3–6 weeks after the last chemotherapy cycle.

### Primary endpoint

The primary efficacy endpoint was the patient self-assessment of hair loss evaluated at least 3 weeks after completing chemotherapy. A Dean's scale score of 0–2 (i.e. HL ≤50%) was considered a success.

### Secondary endpoints

Every patient had to fill specific questionnaires on side effects, adverse events, subjective perception of the device and quality of life, at baseline and after each cycle of chemotherapy. The subjective perception of the device was assessed using the Technology Acceptance Model questionnaire.^9^ It evaluates patients’ attitude toward the new technology and its perceived usefulness. For the assessment of quality of life, the EORT-C30 and EORT-BR23 questionnaires were administered at the beginning of the treatment and at each chemotherapy cycle.

### Statistical analysis

Primary objective was the reduction of HL in at least 55% of patients with a Dean's scale score of 0–2 (i.e. HL ≤50%). Under the assumption that a proportion of success less than 40% indicated insufficient benefit, and that a proportion of success greater than 55% indicated an effective device, a two-stage design^10^ was used in order to allow early termination of the trial. In the first stage, 45 patients were accrued. If at least 20 patients with success had been observed, additional patients were enrolled, leading to a total of 104 patients completing adjuvant chemotherapy. Under the assumption that the true proportion of success was less than 40%, there was a 68% chance of early termination of the study. If the device was actually effective, this two-stage design yielded a probability of 90% of concluding that it is effective. The type I error was set to 5%. The proportion of success was reported with exact binomial 95% confidence interval (CI). Discontinuation of scalp-cooling treatment because of AEs related to the treatment itself or because of alopecia, as well as patient with missing endpoint assessment, were considered as failures. Patient demographic and clinical characteristics at baseline were analysed using descriptive statistics: absolute and relative frequencies for categorical variables and median with range for continuous variables. Concordance between patients’ and physicians’ Dean's scale score was evaluated with Cohen’s Kappa. All analyses were performed using SAS software v. 9.4 (SAS Institute, Cary, NC, USA).

Data on technology acceptance and quality of life (QoL) were analysed on patients that completed the four cycles of adjuvant chemotherapy with anthracyclines using SPSS Package (version 20.0 IBM). Repeated measured ANOVA was used to assess differences in patients’ acceptance and QoL between the baseline and fourth cycle of chemotherapy. The raw scores of the EORTC QLQ-C30 and EORTC QLQ-BR23 were standardised using a linear transformation, obtaining scores ranging from 0–100, where higher scores represent a higher (better) level of functioning (for functional subscales) and higher (worse) level of symptomatology (for symptoms scales). Listwise deletion was used to treat missing values.

## Results

From July 2014 to November 2016, 139 consecutive breast cancer patients were enrolled and provided written informed consent. All the 139 enrolled patients received at least one cycle of chemotherapy with the scalp-cooling system.

Median age was 47 years (range: 28–65), patients were mainly pre- or peri-menopausal (76%), had a ductal histotype (87%) and had nodal involvement in 64%. The more common subtype was Luminal B (60%), 14% of patients had HER2-positive and 8% had triple-negative breast cancer. Patients mainly received anthracyclines-containing adjuvant treatment (95% received at least four cycles of EC) and 32% received the sequence of anthracyclines and taxanes. Patients’ characteristics are shown in Table 1.

**Table 1:** Patients’ characteristics (*N* = 139 enrolled patients)

Characteristic	Levels	*N* (%)
Age, years	<40	29 (21)
	40–49	59 (42)
	≥50	51 (37)
	(median: 47, range: 28–65)	
Menopausal status	Pre and peri	106 (76)
	Post	33 (24)
Histotype	Ductal	121 (87)
	Lobular	5 (4)
	Other	13 (9)
Tumour size	pT1	70 (50)
	pT2-3	68 (49)
	pTx	1 (1)
Positive lymph node	0	49 (35)
	1–3	65 (47)
	>3	23 (17)
	X	2 (1)
Grade	1	0 (0)
	2	57 (41)
	3	81 (58)
	n.e.	1 (1)
Subtype	Luminal A	23 (17)
	Luminal B (Ki67 ≥20%)	84 (60)
	Luminal B (Her-2 positive)	14 (10)
	Her-2 positive	5 (4)
	Triple negative	11 (8)
	Unknown^a^	2 (1)
Type of surgery	Quadrantectomy	58 (42)
	Mastectomy	80 (57)
	Other	1 (1)
Systemic treatment	CT (w or w/o trastuzumab^b^)	18 (13)
	CT (w or w/o trastuzumab^c^) +ET	121 (87)
CT regimen	EC × 4	88 (63)
	EC × 4 + Docetaxel × 4	37 (27)
	EC × 4 + Paclitaxel × 12	7 (5)
	Other	7 (5)

*EC* epirubicin and cyclophpsphamide

^a^Ki-67 missing (both pts were ER/PgR positive, Her-2 negative)

^b^5 pts received trastuzumab

^c^13 pts received trastuzumab

### Primary efficacy analysis

**Fig. 1:**
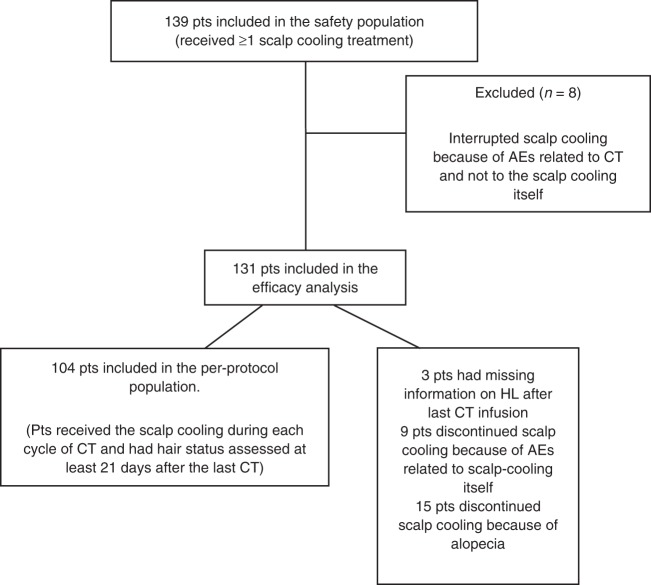
Flowchart of patients in the trial

Eight patients interrupted the scalp-cooling system because of adverse events related to chemotherapy, but not related to the DigniCap® System itself, therefore 131 patients were evaluable and were included in the efficacy analysis (Fig. 1).

Fifty-six out of 131 evaluated patients successfully prevented HL (43%, 95% CI: 34–51%). The remaining 75 patients had treatment failure: 24 patients (32%) discontinued the scalp-cooling treatment because of alopecia observed during treatment (*n* = 15) or scalp-cooling treatment related AEs (*n* = 9), three patients had missing information on the primary efficacy endpoint, and 48 patients (64%) had a HL greater than 50% after completing chemotherapy. Therefore, the results of this trial do not allow to conclude that the scalp-cooling device was effective. One hundred and four patients received scalp-cooling system during all the planned chemotherapy cycles and had their hair status evaluated after the last cycle of chemotherapy (completers population). Among these patients a success in hair preservation was shown in 56 patients (54%, 95% CI: 44–64%). Table 2 shows the chemotherapy regimens received, the duration of treatments and the relative success or failures evaluated at least 21 days after the last chemotherapy infusion. Patient-medical staff agreement in evaluating the grade of alopecia at last chemotherapy infusion indicated a moderate agreement (Weighted kappa: 0.41) and is reported in Table 3.

**Table 2:** Primary efficacy analysis based on patients score 21 days after the last CT infusion

	Treatment success (hair loss ≤50%)	Treatment failures (hair loss >50%)	Total
	*N* (%_row_)	*N* (%_row_)	*N* (%_col_) [median dur]
Overall	56 (54)	48 (46)	104 (100)
CT regimen			
EC × 4	35 (54)	30 (46)	65 (62) [63 days]
EC × 4 + DTX × 4	17 (55)	14 (45)	31 (30) [154 days]
EC × 4 + PTX × 12	1 (50)	1 (50)	2 (2) [143 days]
Other	3 (50)	3 (50)	6 (6) [68 days]

**Table 3:** Patient-medical staff agreement in evaluating the grade of alopecia at last CT infusion

		Nurse/physician score					
Patient score		None	(0–25]	(25–50]	(50–75]	(75–100]	Total
	None	0	0	0	0	0	0
	(0–25]	1	24	1	0	0	26
	(25–50]	0	12	16	2	0	30
	(50–75]	0	10	13	12	0	35
	(75–100]	0	1	5	6	1	13
	Total	1	47	35	20	1	104

Weighted kappa: 0.41, moderate agreement

No. of observed agreements: 53 (51%)

No. of agreement expected by chance: 29 (28%)

### Side effects

All the 139 enrolled patients were included in the safety population analysis. The most common side effects were headaches, discomfort and coldness occurring in 26 patients (19%); three patients had a skin rash, three patients had skin pain and four patients reported dizziness and/or vertigo. No serious adverse event was reported.

### Technology acceptance

Considering patients who completed the study, responses to the TAM (Technology Acceptance Model) Questionnaire showed a general good attitude toward the scalp-cooling system. The results highlighted a higher discomfort in using the device after the first two sessions, with the following mean scores at the end of each treatment cycle: M = 3.60, M = 3.91, M = 4.49 and M = 4.42 (*p* < .01), where 3 = slight comfort, 4 = neither comfort nor discomfort, 5 = slight discomfort. However, this perceived discomfort was not associated with a general decrease in the perceived utility of the device, and the patient’s intention to continue to use it: the mean score indicating patients’ intention to use the scalp-cooling system over the whole period of treatment showed a “high”/“total” agreement with its use (mean scores from the baseline to the fourth cycle of chemotherapy: M = 1.42, 1.64, 1.71, 1.71; *p* = 0.124).

When considering the patients who discontinued the use of the scalp-cooling system, data obtained from their last available measurement showed a significant, even small, reduction in the intention to continue to use the device, compared to patients who continued the use (*p* = 0.012). The mean score reported by patients who decided not to continue after the second cycle was M = 2.28, where 2 corresponded to “high agreement” with the intention to use the device along all the treatment period, and 3 to “slight agreement”. The mean score for patients who continued the study was M = 1.66. For those patients who discontinued, the discomfort was higher than patients who continued the use of the device (M = 4.80 and M = 3.91, respectively, *p* = 0.005).

### Quality of life

Among the patients starting chemotherapy with Dignicap, 58 completed the EORTC-C30 and BR23 questionnaires after 63 days from baseline (fourth treatment cycle). The mean standardised score for perceived General Health Status (GHS) was 70.11 at baseline (SD = 18.47). The mean score of GHS at time 4 was 65.66 (SD = 18.20). The perceived GHS did not significantly change after 63 days, showing a stable good general health status (Table 4).

**Table 4:** Results of the EORTC- QLQ-C30 and EORTC-BR23 reported patients’ breast-related quality of life

	Baseline mean	Fourth cycle mean
*EORTC-C30*		
Global health status	70.1	65.7
Functional subscales		
Physical functioning	93.4	86.1
Emotional functioning	70.7	69.0
Cognitive functioning	90.4	82.8
Social functioning	83.0	73.4
Role functioning	85.7	82.0
Financial difficulties	13.0	12.9
Symptoms subscales		
Fatigue	20.0	34.9
Nausea	1.1	15.0
Dyspnoea	2.7	9.3
Appetite Loss	8.2	10.4
Pain	9.8	12.3
Diarrhoea	0.6	5.8
Constipation	13.6	15.2
*EORTC-BR23*		
Functioning subscales		
Body image	76.9	66.0
Sexual functioning	71.9	78.1
Sexual enjoyment	24.1 (*N* = 18)	46.3 (*N* = 18)
Future perspective	48.9	59.6
Symptoms subscales		
Systemic therapy side effects	6.3	27.0
Breast symptoms	11.8	9.7
Arm symptoms	13.7	11.3
Upset for hair loss	19.4 (*N* = 12)	38.9 (*N* = 12)

Results on the subscales of the EORTC-C30 showed a significant decrease in physical functioning (*p* < .001), cognitive functioning (*p* < .01) and social functioning (*p* < .01), after 63 days from baseline. Fatigue (*p* < .001), nausea (*p* < .001), dyspnoea (*p* < .01) and diarrhoea (*p* < .05) significantly increased after 63 days. No difference was observed in patients’ emotional and role functioning, pain, insomnia, loss of appetite, constipation and financial difficulties.

Results from the EORTC-BR23 showed significantly fewer concerns about their future health (*p* = .05) after 63 days. An increase in systemic therapy side effects was reported (*p* < .001), together with a reduction in perceived patients’ attractiveness (body image; *p* < .01). No difference was found in breast symptoms, arm symptoms and in sexual functioning. However, regarding sexual functioning, patients who reported to be sexually active (*N* = 18) indicated a significant decrease in sexual enjoyment (*p* < .01). Only twelve patients answered the question in the EORTC-BR23 questionnaire on being upset for the hair loss, showing an increased distress over time (*p* < .01).

Annual follow-up is still ongoing and the median follow-up time is 2.4 years (range: 0.8–3.6). All patients are still alive and only two patients had distant relapses with no scalp metastases.

## Discussion

In this prospective study we demonstrated that the scalp-cooling System Dignicap® was able to prevent CIA in 43% of patients who received an adjuvant treatment with anthracycline with or without taxanes, and although this result is lower than originally set as a success (55%), it could be considered as a reasonable option for breast cancer patients undergoing adjuvant chemotherapy for primary breast cancer. If we consider the subgroup of completers population (*n* = 104) the prevention of HL with the scalp-cooling device rises to 54%.

Several studies, particularly in women undergoing treatment for breast cancer, have demonstrated that CIA dramatically worsen patients’ quality of life (QoL).^5,11,12^ In a recent literature review, alopecia was consistently thought of as the most devastating chemotherapy-related adverse effect, resulting in decreased QoL and poor self-image. In fact, some women refuse chemotherapy because of the risk of losing their hair, and in one study,^13^ 8% of women considered refusing chemotherapy, because of the risk of CIA.

Two similar experiences investigating the role of scalp-cooling devices in significantly preventing CIA were recently published in JAMA. Nangia et al. reported on a multicentre randomised clinical trial of women with breast cancer undergoing chemotherapy. Participants were randomszed to scalp-cooling with the Orbis Paxman Hair Loss Prevention System (*n* = 119) or control (*n* = 63). Among the 142 evaluable participants, 36% (*n* = 51) received anthracycline-based chemotherapy and 64% (*n* = 91) received taxane-based chemotherapy. Successful hair preservation was found in 48 of 95 women with cooling (50.5%; 95% CI, 40.7–60.4%) compared with 0 of 47 women in the control group (0%; 95% CI, 0–7.6%). Those who underwent scalp cooling were significantly more likely to have less than 50% hair loss after the fourth chemotherapy cycle compared with those who received no scalp cooling;^14^ however, patients who received anthracyclines had a successful prevention of HL in only 22% of cases. In the same journal Rugo et al. reported on a prospective cohort study of 122 women with stage I or II breast cancer receiving adjuvant or neoadjuvant chemotherapy regimens, excluding sequential or combination anthracycline and taxane (106 patients in the scalp-cooling group and 16 in the control group; 14 matched by both age and chemotherapy regimen). The scalp-cooling device was DigniCap®. HL of 50% or less (Dean's score of 0–2) was seen in 67 of 101 patients (66.3%; 95% CI, 56.2–75.4%) evaluable for alopecia in the scalp-cooling group vs 0 of 16 patients (0%) in the control group (P < .001). Among women undergoing non-anthracycline-based adjuvant chemotherapy for early-stage breast cancer, the use of scalp cooling vs no scalp cooling was associated with less hair loss at 4 weeks after the last dose of chemotherapy.^15^

The results of the present study confirm that the hair-preservation rate with a scalp-cooling system in a population of patients almost completely treated with an anthracyclines-containing chemotherapy is satisfactory and exceeds 40% of successes. There is little data available in the literature regarding patients treated exclusively with anthracyclines, and the available data indicate success rates certainly lower than those reported in the present study. The rate of patients who did not complete the treatment with the support of the device is around 20%, similar to what is found in other analogous studies.^15^

The primary endpoint was based on patient self-assessment, similarly to what reported by Rugo et al.; however, in Nangia et al. the primary efficacy endpoint was assessed by clinicians who were independent and unaware of study treatment. The choice that the primary endpoint is evaluated by the patient instead of the medical staff also reflects the patient’s judgment on the effectiveness or not of the device and - although in some respects it may be a limit - in the present study we noticed that the patient-medical staff agreement in evaluating the grade of alopecia at last chemotherapy infusion indicated a moderate agreement (Weighted kappa: 0.41). Patient scores were reported as generally lower in a 20-patient pilot study using the same scalp-cooling system as used either in the current study and in the study by Rugo.^15,16^

Recently a metanalysis of ten studies included 654 patients. Most were patients with breast cancer, 432 patients (66%) mainly receiving anthracyclines.^17^ For the binary outcome of <50% vs >50% alopecia, the use of scalp cooling reduced relative risk (RR) of alopecia by 43% (RR, 0.57; 95% CI, 0.45–0.72; I2 = 11%; *P* < .00001). For ordinal outcomes (alopecia on a scale of 0–3), use of scalp cooling significantly reduced alopecia (MD, −0.80; 95% CI, −1.19 to −0.41; I2 = 0%; *P* < .0001). This systematic review and meta-analysis support the use of scalp cooling to prevent alopecia in patients with solid tumours undergoing chemotherapy.

The present trial represents the first experience with a scalp-cooling device including patients (95%) almost exclusively treated with anthracyclines. Cooling cap system is generally well-tolerated.^18^ In our series the percentage of dropout due to Scalp Cooling System was quite low, nine patients (7%) among 139 enrolled did not tolerate Dignicap® and discontinued the study. In the trial by Nangia et al. dropout for discomfort was around 6%.^14^ One concern is about satisfactory HL prevention, because 11% of the patients were not satisfied and interrupted the scalp-cooling system. These patients contribute to the 57% who had a treatment failure. The HL was mainly in the vertex in the area known as “mid-scalp”, the area of the skull between the parietal and the temporal bone, delimited laterally by the temporal and parietal fringes. A possible explanation is that this area has a rich vascularisation of the scalp with the characteristic centripetal organisation with vessels running in the subcutaneous connective tissue. The fit of the cap is key to successful hair retention with the scalp-cooling devices, and there is a learning curve with use of the device that must be considered when the nurse personnel change, as they need a certain period to become more skilled at ensuring a tight fit. This aspect was quite common in other similar trials and was reported as potential limit in the results.^14^ In our opinion the challenges to improve the outcome of reduction in hair loss are related to the best possible fit of the cooling cap, the maintenance of an adequate temperature (3°–5 °C) and patient’s compliance.

The most common reported side effects were headaches, unpleasant feelings due to the heaviness of the cap and coldness, dizziness and transient light-headedness. The general health status reported by patients was good from the beginning of the chemotherapy until the end of the fourth cycle.

Patients’ main functions decreased during the four cycles, especially the physical, cognitive and social ones. Emotional functioning remained stable over time. However, the level of these functions was on average still good or very good.

Only twelve patients answered the question in the EORTC-BR23 questionnaire on being upset for the HL, showing an increased distress over time. The results on the TAM questionnaire, however, showed that, despite the side effects and the increase in discomfort using the scalp-cooling device, patients considered it an important tool to be used and showed a stable intention to use it. Among the patients who discontinued the use of the scalp-cooling system, there was an even higher increase in discomfort. This might have affected the perception of positivity of the device and therefore their decision to interrupt. According to the affective heuristic, the negative sensation attached to the experience positively correlates with the probability to avoid that experience.^19^

DigniCap® system determines a reduction of blood perfusion to scalp. A possible consequence is risk of scalp metastases, as result of the decreased drug exposure. The primary concern that has limited the use of scalp-cooling devices in the United States is the possibility that scalp cooling could increase the risk for scalp metastases, although they are a rare site of metastatic disease in breast cancer.^20,21^ This concern was evaluated by Grevelman and Breed in a recent review:^7^ they analysed 56 scalp-cooling studies founding only nine patients with scalp skin metastases among approximately a total of 2500 patients. In all these cases, it was very unlikely that scalp metastases were a result of scalp cooling. Scalp metastases were never the first single site of recurrence, but were diagnosed at the same time, or after a previous diagnosis of metastases in other sites. A recent review of the literature on scalp metastasis following adjuvant chemotherapy for early-stage breast cancer^22^ found it unlikely that the incidence of scalp metastasis might increase after scalp cooling. None of our patients treated with DigniCap® System developed scalp metastases after a median follow-up of 2.4 years, and although the follow-up is still short we will continue to follow these patients.

This study has several limitations, including monocentric evaluation and the not randomised type of trial. However, since other studies have been published using the DigniCap® system and all have shown a significant advantage in using the device for CIA prevention, it may be unethical to randomise a group of patients to receive the scalp-cooling system or not. Nevertheless, another concern is the cost and time-spending, because the use of Dignicap® System requires a dedicated nurse for additional time in the infusion room. There are variable costs of the devices depending on the duration of chemotherapy and the type of cap used, but the average total cost for scalp cooling is estimated to range between $1500 and $3000 per patient depending on the number of treatment cycles. In addition, there may be institutional costs associated with extra time in the chemotherapy infusion centre and additional personnel costs.^23^

## Conclusion

In our series DigniCap® System was able to prevent significantly HL in more than 40% of primary breast cancer patients receiving a planned adjuvant chemotherapy with anthracyclines. DigniCap® System could be integrated in supportive care of breast cancer patients. Longer follow-up is needed to assess long-term safety.

## Data Availability

Data supporting the results reported in the article are available at Clinical Trial Office, European Institute of Oncology, Milan, Italy. Restrictions apply to the availability of these data, which were used under license for the current study, and so are not publicly available. Data are, however, available from the authors upon reasonable request and with permission of European Institute of Oncology, Milan, Italy.
